# Dutcher bodies as a diagnostic key to POEMS syndrome

**DOI:** 10.1002/jha2.656

**Published:** 2023-02-07

**Authors:** Ikuo Matsuda, Seiichi Hirota

**Affiliations:** ^1^ Department of Surgical Pathology Hyogo Medical University School of Medicine Nishinomiya‐city Japan

**Keywords:** Dutcher bodies, neoplasm, plasma cell, POEMS syndrome

1

A 55‐year‐old Japanese man was presented to our hospital for examination of edema in the lower legs, followed by numbness in the lower legs and penis, and erectile dysfunction for the past 4 months. Physical examination showed hypoesthesia of the bilateral feet, indicating peripheral polyneuropathy. Imaging studies showed pleural and pericardial effusions. Blood tests revealed high levels in leukocyte (108.2 × 10^2^ /µl) and platelet (63.7 × 10^4^ /µl) counts, C‐reactive protein (1.27 mg/dl), potassium (7.2 mmol/L), blood urea nitrogen (82 mg/dl), and creatinine (2.22 mg/dl). Acute kidney injury was suggested. Computerized tomography of the sagittal section of the body showed multiple osteosclerotic lesions in the vertebrae (Figure 1A, arrowheads). A metastatic bone tumor was suspected, although tumor markers and history presented no evidence of the primary tumor. A biopsy of the osteosclerotic lesions was performed. Hematoxylin‐eosin image of the bone biopsy showed a few areas of tiny cellular nests among sclerotic bones at low magnification (Figure 1B, circles; bar: 200 µm). A careful examination of the cells in the nests at high power revealed the presence of nuclear pseudoinclusion called Dutcher bodies (Figure 1C, circles; bar: 20.0 µm), suggesting their plasma cell origin. Immunohistochemistry showed that the lesion was positive for CD138 and negative for cytokeratin AE1/AE3. In situ hybridization for immunoglobulin (Ig) κ and Ig λ confirmed that the tumor was an Ig λ‐positive plasma cell neoplasm. Taken together, the diagnosis was established as POEMS syndrome, characterized by polyneuropathy (“P”), organomegaly (“O”), endocrine abnormality (“E”), monoclonal proliferation of plasma cells (“M”), and skin lesion (“S”). [1] The present case meets its one major criteria (osteosclerotic bone lesions) and two minor criteria (extravascular volume overload and thrombocytosis) in addition to “P” and “M”. The patient underwent Rd (lenalidomide and dexamethasone) therapy, followed by high‐dose melphalan administration and autologous peripheral blood stem cell transplantation. Four and a half years later, a bone marrow aspiration sample was negative for minimal residual disease by 8‐color flow cytometry. In the present case, a plethora of clinical findings hampered a unified interpretation, and hence, an early diagnosis. A pathologist's eye integrated a histological clue (Dutcher bodies) and all clinical findings, leading to a definite diagnosis.

**FIGURE 1:**
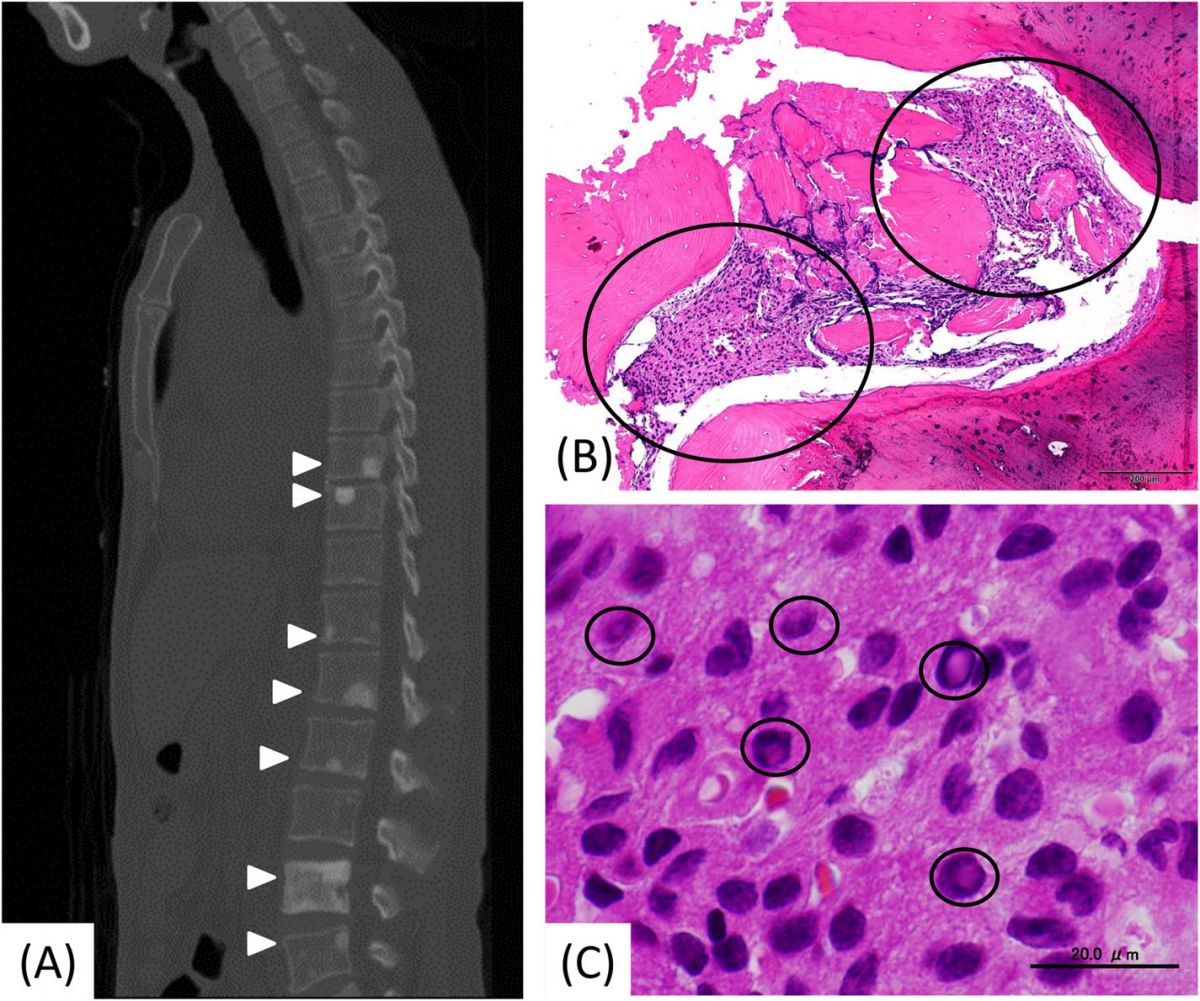
(A) Computerized tomography showed multiple osteosclerotic lesions (arrowheads) in the vertebrae in the sagittal section. (B) Hematoxylin‐eosin image of the bone biopsy at low magnification (bar: 200 µm). Tumor cell nests were enclosed by circles. (C) Dutcher bodies were enclosed by circles. Original magnification: x1000. Bar: 20.0 µm.

## CONFLICT OF INTEREST STATEMENT

The authors declare no conflict of interest.

## ETHICS STATEMENT

This work was approved by the Ethics Review Board of Hyogo Medical University (No.0231).

## PATIENT CONSENT STATEMENT

Informed consent was obtained from the patient for the publication of this article.

## Data Availability

The data that support the findings of this study are available at reasonable request from the corresponding author. The data are not publicly available due to privacy or ethical restrictions.
